# Efficacy of carbonic anhydrase inhibitors in management of cystoid macular edema in retinitis pigmentosa: A meta-analysis

**DOI:** 10.1371/journal.pone.0186180

**Published:** 2017-10-12

**Authors:** Qinzhu Huang, Ru Chen, Xianping Lin, Zhenyang Xiang

**Affiliations:** Taizhou Hospital, Wenzhou Medical University, Taizhou, Zhejiang, PR China; Universita degli Studi di Firenze, ITALY

## Abstract

**Background:**

Carbonic anhydrase inhibitors (CAI) are often used in the treatment of cystoid macular edema (CME) in retinitis pigmentosa (RP) patients. The aim of this meta-analysis is to gain a better understanding of the overall efficacy of CAI treatment.

**Methods:**

Databases including PubMed, EMBASE, and Cochrane Library were searched to identify relevant studies. Eligible studies were clinical trials of patients with RP assigned topical or oral CAIs such as dorzolamide and acetazolamide. Changes in central macular thickness (CMT) by OCT in μm and best-corrected visual acuity (BCVA) in log MAR equivalents were extracted and results compared between baseline and after treatment.

**Results:**

11 clinical reports were identified which included a total of 194 patients (358 eyes) available for analysis, with 59 patients (115 eyes) assigned oral CAI treatment and 135 patients (243 eyes) assigned topical CAI treatment. The combined results showed a significant reduction of macular edema, as calculated by baseline and final central macular thickness (CMT) based on OCT examination (46.02μm, 95%CI: -60.96, -31.08, *I*^*2*^ = 65%). However, the effect on visual acuity was inconsistent across studies.

**Conclusion:**

Based on non randomized controlled clinical studies, RP patients with CME who were treated with CAIs had better anatomical outcomes, but the effect on visual acuity was contradictory across studies. Multicenter prospective randomized controlled trials would be ideal to definitively test its clinical efficacy in RP patients.

## Introduction

Retinitis pigmentosa (RP) is a heterogeneous group of inherited retinal disorders. According to the inheritance pattern, it is usually classified into three subtypes: autosomal dominant, autosomal recessive, and X-linked forms. There are specific forms of RP such as Usher syndrome, which is characterized by congenital sensorineural hearing loss in conjunction with RP [1]. Clinical symptoms of RP patients include night blindness and progressive visual field loss resulting from degeneration of photoreceptors, which eventually leads to blindness. Complications such as an epi-retinal membrane, cataracts, or cystoid macular edema (CME) can can also cause early visual loss. According to clinic-based surveys, the prevalence of CME in patients with RP ranges from 11% to 49% [2,3,4,5,6,7]. The wide variation could be partly explained by the resolution quality of various examination methods such as ophthalmoscopy, fluorescein angiography, and optical coherence tomography (OCT).

A number of interventions have been applied to treat CME in RP. Reports show that RP patients with CME may benefit from the administration of reagents such as CAI’s [8,9], intravitreal anti-vascular endothelial growth factor (VEGF) agents [10,11], and intravitreal corticosteroids [12,13,14,15]. Among these therapies, both topical and oral CAIs have been reported to be useful in managing CME. However, the majority of reports are relatively small cases series, efficacy rates vary greatly between different groups [16,17,18], and visual acuity (VA) improvements after treatment are still uncertain. To our knowledge, there has been no systematic review significant enough to evaluate the potential of CAI treatment. Therefore, we undertook a meta-analysis to assess the efficacy of CAI for the management of CME in RP.

## Methods

### Literature search

We conducted searches of the following electronic databases: PubMed, Cochrane Library, and Embase without language restriction. We used the combinations of the following terms: carbonic anhydrase inhibitors, ethoxzolamide, acetazolamide, dorzolamide, pigmentary petinopathy/pigmentary retinopathies, retinopathies pigmentary/retinopathy pigmentary, retinitis pigmentosa, and macular edema. The search strategy for PubMed can be found in Supporting Information (**S2 File**). In addition, we manually screened the pending references of original reports to identify studies not yet included in the previous literature search. If sequential reports from one group which investigated the same cohort of patients were identified, only the latest updated or informative one was included. The final search was carried out on October 2016.

### Selection criteria

Articles selected from this initial search were considered eligible for inclusion in the meta analysis using the following criteria: (1) study design: Randomized Controlled Trials (RCTs), Non-randomized comparative studies such as single-arm studies, cross-over studies and retrospective cohort studies; (2) population: RP patients with CME; (3) intervention: topical and oral CAI; (4) outcome variables: baseline and mean stopping VA or the central macular thickness (CMT) data obtained by OCT was included. Reports were excluded using the following criteria: (1) full texts and abstracts from conferences without raw data; (2) duplicate publications; (3) letters, comments, and reviews; (4) subjects were of rebound macular edema; (5) patients receiving multiple treatments.

### Data extraction

Two reviewers extracted data independently. Disagreement was resolved by discussion on all items. The following information was extracted from the original studies: first author of each study, publication year, information on study design, number of patients/eyes, sex, intervention, mean age, VA, and CMT measured by OCT. If the trials reported raw data including all phases of follow-up, only data from the last follow-up time were analyzed.

### Quality assessment

Quality assessments were conducted independently by two authors, and disagreements were resolved by discussion. RCTs were assessed using a Jadad scale, [19] while single arm studies and cross-over studies and retrospective cohort studies were evaluated using the Newcastle Ottawa Scale (NOS).[20] For Jadad scale, studies scoring 3 points were considered to be of low quality. For the NOS scale, overall study quality was defined as poor (score, 0–3), fair (score, 4–6) or good (score, 7–9).

### Statistical analysis

All data was analyzed using Review Manager (RevMan [Computer program], Version 5.3, Copenhagen: The Nordic Cochrane Centre, The Cochrane Collaboration, 2014).The main outcome variables were VA and CMT measured by OCT. If the VA was reported in Snellen VA form in original publication, the data was converted to logarithm of minimal angle of resolution (logMAR) for statistical analysis. Based on a previous report, “counting fingers” (CF), “hand motion” (HM), “light perception” (LP), and “no light perception (NLP)” were assigned a logMAR value of 1.85, 2.3, 2.7 and 3.0 respectively [21,22]. The mean changes of VA or CMT from baseline to final follow-up points were pooled and calculated using inverse variance methods. Both outcomes were reported with a 95% confidence interval (CI). P<0.05 was considered statistically significant. Heterogeneity was analyzed with the *I*^*2*^ statistic, and defined as low (25% to 50%), moderate (50% to 75%), or high (>75%) [23]. Subgroup analyses were conducted based on follow-up period, intervention, sample size and baseline level. The *I*^2^ value > 50% was defined as heterogeneity and a random-effects model was applied to the data. Otherwise, a fixed effects model was considered for pooling the data.

## Results

### Overall characteristics of selected trials and quality assessment

**Fig 1:**
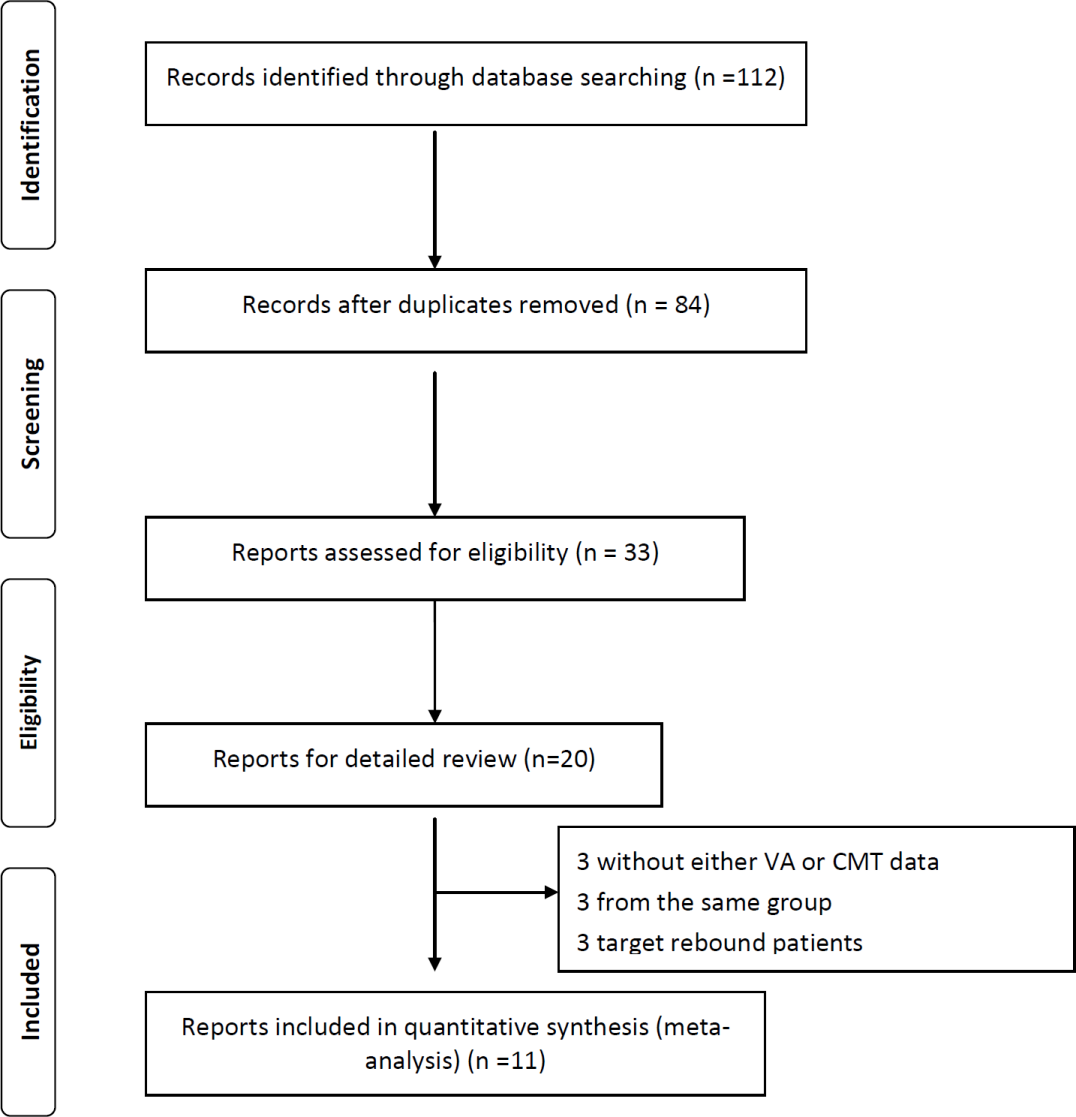
Flow chart of trials included in this meta-analysis.

A total of 112 reports were initially identified. Of these, 101 were excluded based on the exclusion criteria listed above. The 11 remaining clinical reports (1 abstract and 10 full-text) that met the inclusion criteria were analyzed [8,9,16,18,24,25,26,27,28,29,30]. **Fig 1**provides a flow chart of the search results. These 11 reports include 1 randomized controlled trial (RCT); 2 retrospective cohort studies, 2 cross-over studies and 6 before-after trials. The RCT study was not pooled and kept separate in the meta-analyses. If classified with intervention, 1 has both oral and topical CAI groups; 5 are treated with oral CAIs; the other 5 are treated with topical CAIs. In total, there were 358 eyes of 194 patients included in this meta-analysis. 59 patients (115 eyes) were included in the oral CAIs group, and 135 patients (243 eyes) were included in the topical CAIs group. In addition, the overall quality scores of the included studies were presented in Table 1, with the exception of one study only with English abstract.

**Table 1:** Characteristics of included trials and patients.

First author	Year	Study design	Patients (eyes)	Intervention	VA (logMAR)		Central Macular Thickness (CMT, μm)		Quality scoring
					Mean difference	Standard error	Mean difference	Standard error	
Liew[24]	2015	Retrospective cohort study	64 (115)	Dorzolamide, topical	NR	NR	-46	7	5
			17 (32)	Acetazolamide, oral	NR	NR	-37	12	
Alkin[25]	2013	Retrospective cohort study	6 (8)	Brinzolamide, topical	0.08	0.05	-42	10	**/**
Reis[26]	2015	RCT*	9 (13)	Dorzolamide, topical	-0.06	0.04	-24	15	4
Orzalesi[27]	1993	Single arm trial	7 (14)	Acetazolamide, oral	-0.12	0.02	NR	NR	4
Chung[28]	2006	Single arm trial	10 (20)	Acetazolamide, oral	-0.08	0.04	-96	24	7
Ikeda[18]	2013	Single arm trial	9 (16)	Dorzolamide, topical	0.11	0.05	-71	14	6
Genead[16]	2010	Single arm trial	32 (64)	Dorzolamide, topical	-0.08	0.02	-18	8	5
Moldow[29]	1998	Cross-over study	9 (17)	Acetazolamide, oral	0.02	0.01	NR	NR	5
Fishman[9]	1989	Cross-over study	12 (24)	Acetazolamide, oral	-0.08	0.02	NR	NR	6
Grover[30]	2006	Single arm trial	15 (27)	Dorzolamide, topical	-0.07	0.02	-49	15	5
Cox[8]	1988	Single arm trial	4(8)	Acetazolamide, oral	-0.31	0.07	NR	NR	3

*The control group is Ketorolac.

RCT: Randomized controlled trial, NR: Not reported

### Efficacy analysis

**Fig 2:**
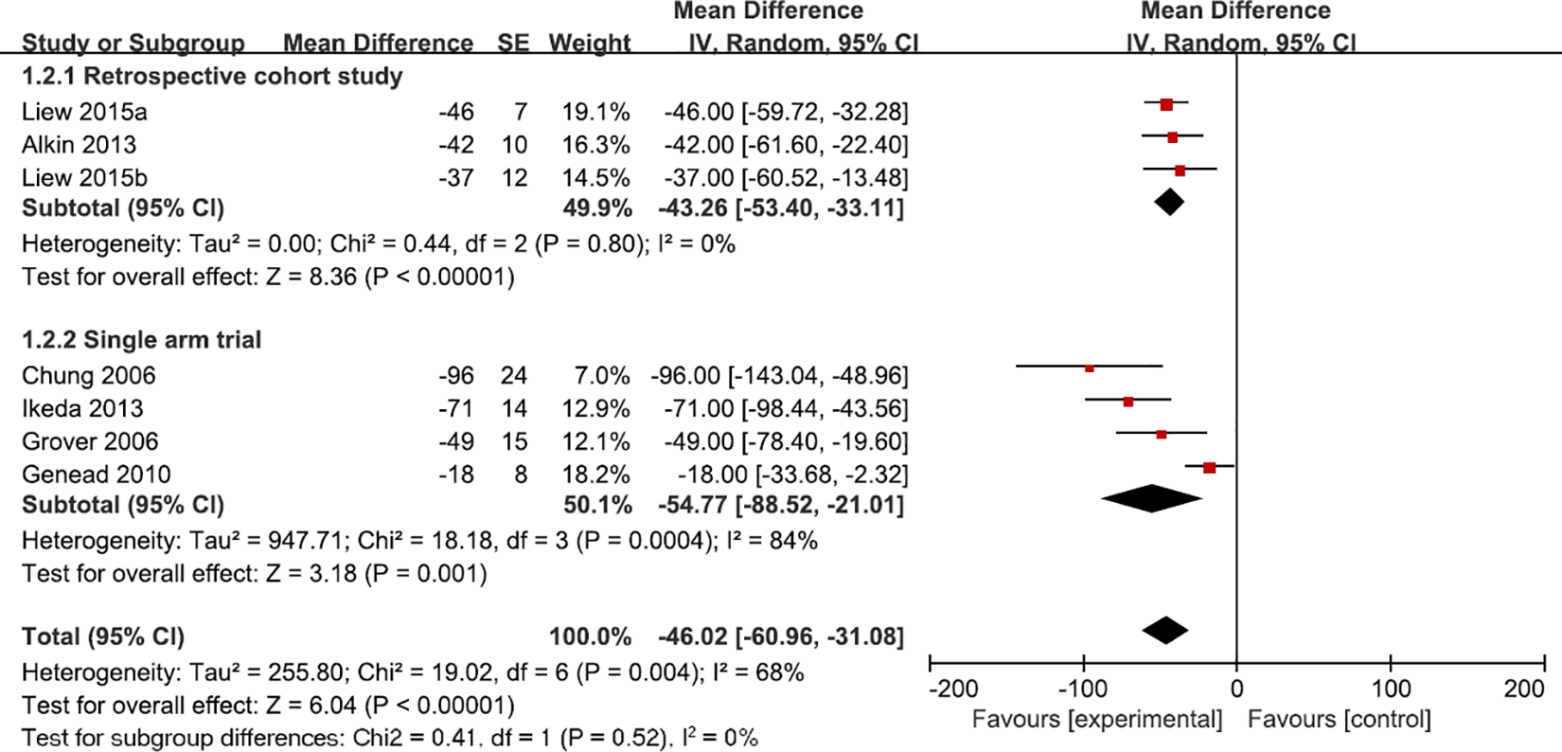
Forest plot shows the mean change of central macular thickness (CMT) from baseline. Data Liew 2015a (topical CAI group) and Liew 2015b (topical CAI group) were extracted from one report. SE = standard error; IV = inverse variance; CI = confidence interval.

**Fig 3:**
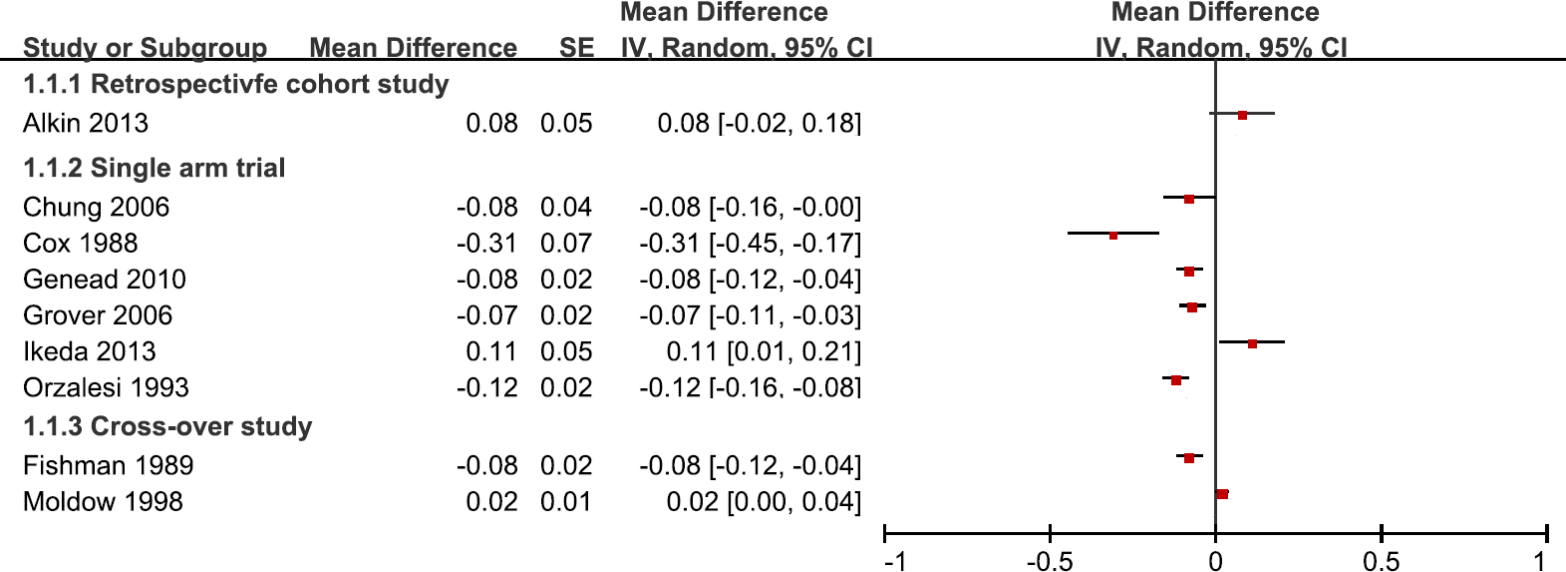
Diagram shows the 95% CI of mean change of best corrected visual acuity from baseline in each study.

Data for the therapeutic effect of treatment with CAIs in RP patients with CME were available for pooled analysis. The short-term treatment with CAIs was 3 weeks and the long-term treatment was 58months [16,18,27]. The combined results showed reduction of macular edema through OCT examination. The pooled mean difference in central macular thickness (CMT) reduced 46.02μm (95% CI: -60.96, -31.08, *I*^*2*^ = 65%) from baseline to the final follow-up points (**Fig 2**). However, when we tried to combine all visual acuity results, it was found that the I-square was as high as 91%. Thus, we gave up on conducting such a meta-analysis. **Fig 3**shows the 95% CI of each study. With most studies favoring CAIs treatment, there were three studies that gave contradictory estimates. Altogether, these analysis shows that CMT decreases with CAIs treatment, but the effect on visual acuity was inconsistent across studies.

### Subgroup analyses

**Fig 4:**
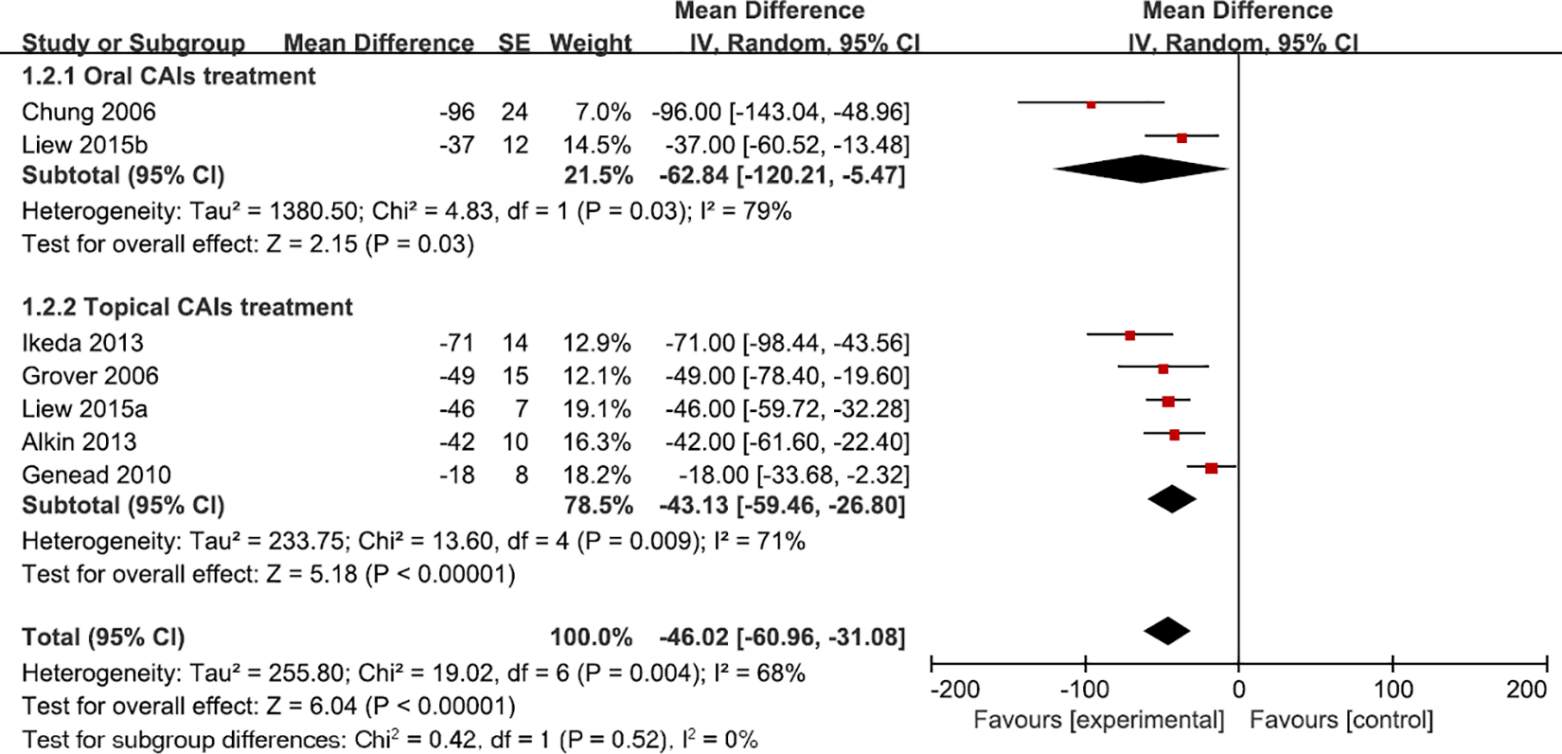
Forest plot shows subgroup analysis of CMT according to interventions (Topical VS Oral).

**Fig 5:**
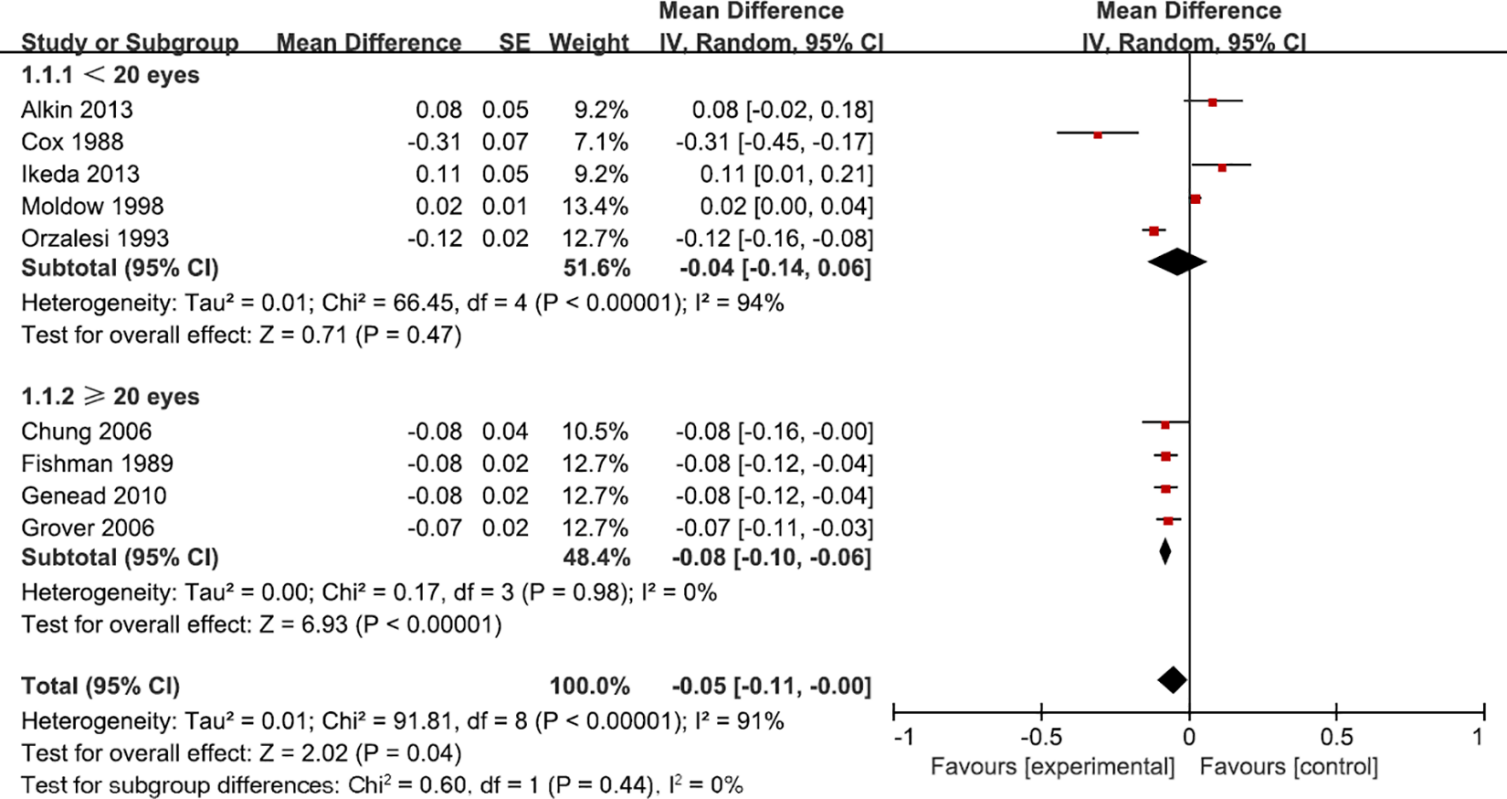
Forest plot shows subgroup analysis of VA based on sample size.

We conducted subgroup analysis to explore the source of heterogeneity in CMT and VA with respect to follow-up period, intervention, sample size and baseline level. More CMT change was observed in long term follow-up studies (mean difference = -58.25; 95% CI: -105.78, -10.72), oral Acetazolamide (mean difference = -62.84, 95% CI: -120.21, -5.47) (**Fig 4**), studies of 20 less sample size (mean difference = -54.84, 95% CI: -83.08, -26.61) and studies with higher CMT baseline level (mean difference = -48.60, 95% CI: -60.43, -36.77). In the subgroup analysis of mean VA difference, we found that the substantial heterogeneity still remained (**Table 2**). One clue from the VA subgroup analysis is that studies with larger sample sizes provide more stable results (mean difference = -0.08, 95% CI: -0.10, -0.06) (**Fig 5**). Other forest plots for subgroup analysis can be found in supplementary materials (**Fig A-D in S3 File**).

**Table 2:** Subgroup analysis to explore the source of heterogeneity on changes in CMT and VA.

CMT			VA		
subgroup	Mean difference (95% CI, μm)	*I*^*2*^	subgroup	Mean difference (95% CI)	***I***^***2***^
follow-up period			follow-up period		
≤4 mo	-43.87 (-53.45, -34.28)	0	≤3 mo	-0.06 (-0.15, 0.04)	93
>4 mo	-58.25 (-105.78, -10.72)	89	>3 mo	-0.06 (-0.11, -0.01)	79
intervention			intervention		
Topical	-46.25 (-65.61, -26.89)	75	Topical	-0.00 (-0.08, 0.07)	85
Oral	-62.84 (-120.21, -5.47)	79	Oral	-0.10 (-0.18, -0.02)	94
sample size			sample size		
<20	-54.84 (-83.08, -26.61)	65	<20	-0.04 (-0.14, 0.06)	94
≥20	-42.75 (-61.30, -24.21)	71	≥20	-0.08 (-0.10, -0.06)	0
baseline			baseline		
<360	-44.77 (-77.15, -12.38)	82	>0.5	-0.05 (-0.13, 0.02)	85
≥360	-48.60 (-60.43, -36.77)	17	<0.5	-0.06 (-0.15, 0.03)	96

## Discussion

CME is a common manifestation of various eye diseases such as diabetic retinopathy, retinal vein occlusion, chronic uveitis, and retinitis pigmentosa. Given the importance of macular function in vision, the treatment of CME can never be over emphasized. Fortunately, CME is treatable and can be alleviated by intravitreal corticosteroids, anti-VEGF agents, and other medications. In this meta-analysis we reviewed studies that treated CME in RP patients with topical or oral CAI’s. The combined results showed a significant reduction in central macular thickness after CAIs treatment (46.02 μm, 95% CI: -60.96, -31.08, *I*^*2*^ = 65%). The pooled VA data indicate that most studies (with the exception of 3 studies) show improvements in VA after CAIs treatment (**Fig 3**). In one RCT report, CAIs treatment in PR patients achieved significant improvement in the first 6 months, however a regression was observed at 1 year (Table 1) [26]. Ikeda *et al* recommended Humphrey Field Analyzer as a more sensitive technique to evaluate macular sensitivity in RP patients with CME [17]. It is proposed that reduced VA may be due to macular cell loss or irreversible functional damage during the development of edema [17,28], thus it’s difficult to recover. Rebound is always an issue when dealing with CME, however, RP patients who show signs of recurring macular edema can restart the CAIs treatment after a period of discontinued use and still have a favorable response [31].

The exact mechanism of how CAIs reduce CME in RP patients remains unclear. Two working models have been proposed. Some authors have proposed that CAIs restore a relatively normal distribution of carbonic anhydrase activity and polarity of RPE cells by selectively blocking the activity of different anhydrase isozymes located in basolateral membrane of RPE cells, thus facilitating subretinal fluid resorption [8,32]. However, some studies suggest a direct role of CAIs on the retinal vasculature as they show that CAIs are capable of enhancing retinal blood flow and oxygen tension [33,34].

It has been reported that systemic CAIs can cause side-effects such as appetite loss, fatigue, and even the development of kidney stones [35]. However, the side effects rate was low in the published reports, and it could be reduced by a lower yet effective dosage (125 mg/d) [28]. Compared to oral CAIs, the side effects during topical administration were minimal. The most common side-effect was a short-term stinging sensation in the eye immediately after instilling [30]. Allergic blepharitis can be avoided by using preservative-free eye-drops [26].

Several limitations of this work are worth considering. First, we cannot fully exclude publication bias. We only searched literature with English abstracts. Second, results are affected by the quality of individual studies. These studies were carried out with small sample sizes and had different duration of intervention, populations, and different follow-up period. Third, due to the paucity of RCTs, this review primarily evaluated cohort studies and single-arm studies, thus associations between intervention and outcomes are at some risk for bias, which is a source of heterogeneity. The possibility of CME alleviation by itself during long follow-up period also cannot be fully excluded.

In conclusion, the current meta-analysis has shown that treatment of CME in RP patients with CAIs significantly reduces the central macular thickness, but the effects on visual acuity are contradictory across studies. Thus, multicenter prospective randomized controlled trials would be ideal to definitively test its clinical efficacy in RP patients.

## Supporting information

S1 File PRISMA checklist. (DOC)

S2 File Search strategy for PubMed. (DOCX)

S3 File Supplementary figures. (DOCX)
