# Supplementary material for: Efficacy of carbonic anhydrase inhibitors in management of cystoid macular edema in retinitis pigmentosa: A meta-analysis
Source: PLoS One. 2017 Oct 12;12(10):e0186180. doi: 10.1371/journal.pone.0186180 (PMC5638411; doi:10.1371/journal.pone.0186180)
Supplement: S2 File — (DOCX) [file pone.0186180.s002.docx]

**Search strategy for PubMed**

Full details of the search strategy regarding PubMed are:

(((((("Carbonic Anhydrase Inhibitors"[Mesh]) OR "Ethoxzolamide"[Title/Abstract]) OR "Acetazolamide"[Title/Abstract])) OR dorzolamide[Title/Abstract])) AND ((((((((Pigmentary Retinopathy[Title/Abstract]) OR Pigmentary Retinopathies[Title/Abstract]) OR Retinopathies, Pigmentary[Title/Abstract]) OR Retinopathy, Pigmentary[Title/Abstract])) OR "Retinitis Pigmentosa"[Mesh])) AND Macular Edema[MeSH Terms])
