# Supplementary material for: Efficacy of carbonic anhydrase inhibitors in management of cystoid macular edema in retinitis pigmentosa: A meta-analysis
Source: PLoS One. 2017 Oct 12;12(10):e0186180. doi: 10.1371/journal.pone.0186180 (PMC5638411; doi:10.1371/journal.pone.0186180)
Supplement: S3 File — (DOCX) [file pone.0186180.s003.docx]

**Supplementary figures**

**Fig A. Forest plot shows subgroup analysis of CMT according to follow-up duration.**

**Fig B. Forest plot shows subgroup analysis of CMT according to sample size.**

**Fig C.Forest plot shows subgroup analysis of CMT according to baseline**

** Fig D. Forest plot shows subgroup analysis of VA according to follow-up duration.**

**Fig. S5 Forest plot shows subgroup analysis of VA according to interventions.**

**Fig. S6 Forest plot shows subgroup analysis of VA according to baseline.**
